# Draft Genome Sequences of Five New Strains of Methylophilaceae Isolated from Lake Washington Sediment

**DOI:** 10.1128/genomeA.01511-14

**Published:** 2015-02-05

**Authors:** Tami L. McTaggart, Gabrielle Benuska, Nicole Shapiro, Tanja Woyke, Ludmila Chistoserdova

**Affiliations:** a Department of Chemical Engineering, University of Washington, Seattle, Washington, USA; b DOE Joint Genome Institute, Walnut Creek, California, USA

## Abstract

We sequenced the genomes of five new Methylophilaceae strains isolated from Lake Washington sediment. We used the new sequences to sort these new strains into specific Methylophilaceae ecotypes, including one novel ecotype. The new genomes expand the known diversity of Methylophilaceae and provide new models for studying the ecology of methylotrophy.

## GENOME ANNOUNCEMENT

We recently described the diversity of Methylophilaceae from Lake Washington through an analysis of the genomes and the phenotypes of 11 strains, separating them into eight distinct ecotypes: Methylotenera mobilis JLW8, M. mobilis 13, Methylotenera versatilis 301, Methylovorus glucosotrophus, Methylophilaceae 7, Methylophilaceae 11, Methylophilus methylotrophus Brown, and M. methylotrophus White (1). Here, we report the draft genome sequences of five additional strains from Lake Washington sediment, isolated using the techniques described by Beck et al. (1). Strain 7 was isolated from the 2011 sample (1), strain G11 was isolated from the 2013 sample (2), strain L2L1 was isolated from long-term methane enrichment (2), and strains Q8 and N17 were isolated from short-term methane enrichment (2).

The draft genomes were generated at the Department of Energy (DOE) Joint Genome Institute (JGI) using Pacific Biosciences (PacBio) sequencing technology (3) and assembled using HGAP (version: 2.2.0.p1) (4), as part of the JGI sequencing pipeline (http://www.jgi.doe.gov). The genome statistics are shown in Table 1.

**TABLE 1:** Strains described, closest relatives, genome statistics, accession numbers, and variable metabolic modules

Strain	Closest relative	% 16S rRNA gene identity	% proteins with >80% identity	Total no. of base pairs	NCBI accession no.	Presence of^*a*^:					
						MxaFI	MADH	NMGP	NarABC	NirK	NorBD
Q8	M. methylotrophus 1	100	86.4	2,900,053	JUHE00000000	+	+	−	−	−	−
L2L1	M. mobilis 13	99.6	79.3	2,641,989	JQMG00000000	+	−	−	+	+	+
N17	Methylophilaceae 11	99.9	88.5	2,753,666	JUGE01000001	+	−	−	−	−	−
7	Methylophilaceae 7	97.3	69.1	2,503,174	JUGF00000000	+	+	−	−	−	−
	Methylophilaceae G11	97.6	21.3	2,503,174	JUGF00000000	+	+	−	−	−	−
G11	M. versatilis 301	98.5	21.2	2,545,099	JUHH01000001	−	+	+	−	+	+
	M. mobilis JLW8	97.5	37.0	2,545,099	JUHH01000001	−	+	+	−	+	+

a MxaFI, calcium-containing methanol dehydrogenase; MADH, methylamine dehydrogenase; NMGP, *N*-methylglutamate pathway; NarABC; NirK, dissimilatory nitrate and nitrite reductases; NorBD, nitric oxide reductase.

Strain Q8 is most similar to M. methylotrophus sp. strain 1, and strain L2L1 is most similar to M. mobilis 13 (Table 1). The main difference in metabolic potential is the lack of the gene for nitrous oxide reductase in the genome of strain L2L1. Strain N17 is most similar to Methylophilaceae sp. strain 11 (Table 1). Strain 7 does not have close relatives; at the 16S rRNA gene identity level, it is related most closely to strain G11 and less related to the Methylophilaceae 7 ecotype. However, in terms of genome-genome similarity, this strain is more related to Methylophilaceae 7 (Table 1). Thus, we ascribed this strain to the Methylophilaceae 7 ecotype. Phenotypically, strain 7 is similar to other strains within this ecotype. However, it has a somewhat enhanced methylotrophy potential, encoding an additional XoxF enzyme and a true Fae that is missing from strains 73s and 79 (1). The protein encoded by this gene is most related to a Fae in the M. glucosotropus SIP3-4 genome (83%) (5), suggesting a horizontal transfer event. At 2,503 Mb, this genome is the smallest so far for Lake Washington Methylophilaceae.

Strain G11 is the most novel strain of the set. In terms of 16S rRNA gene identity, it is most similar to M. versatilis 301, while in terms of genome-genome similarity, it is somewhat more related to M. mobilis JLW8 (Table 1). Thus, we identify this strain as a novel ecotype within Lake Washington Methylophilaceae. Like strains JLW8 and 301, G11 does not encode calcium-containing (MxaFI) methanol dehydrogenase but encodes three XoxF-type proteins. Like strain JLW8, it encodes methylamine dehydrogenase, and like strain 301, it encodes the *N*-methylglutamate pathway for methylamine oxidation (1).

The availability of these new genomes further expands the diversity of Methylophilaceae from a single ecological niche and further points toward unexpected metabolic flexibility within this taxon. The new strains described here also expand the repertoire of model organisms that can be used to study the ecology of methylotrophy.

### Nucleotide sequence accession numbers.

The sequences have been deposited in GenBank under the accession numbers listed in Table 1.
